# Effects of Palatal Expansion with Torque Activation using a Transpalatal Arch: A Preliminary Single-Blind Randomized Clinical Trial

**DOI:** 10.1155/2021/8883254

**Published:** 2021-06-01

**Authors:** Fataneh Ghorbanyjavadpour, Vahid Rakhshan

**Affiliations:** ^1^Orthodontics Department, School of Dentistry, Ahvaz Jundishapur University of Medical Sciences, Ahvaz, Iran; ^2^Department of Dental Anatomy, Dental Branch, Azad University, Tehran, Iran

## Abstract

**Purpose:**

The literature regarding the treatment of posterior crossbites using a transpalatal arch (TPA) is scarce. Moreover, there is only one clinical study on the correction of unilateral crossbites using torque activation. This is an important clinical issue; therefore, this study was conducted to show the effects of an active Goshgarian TPA in correcting nonfunctional single-tooth unilateral crossbite.

**Methods:**

The present single-blind, randomized clinical trial examined 60 observations on 30 individuals with nonfunctional single-tooth unilateral crossbites in the first permanent molar area. Patients were randomly divided into two groups of “symmetric expansion” [control] and “expansion + torque activation” using Goshgarian TPAs [experimental]. The palatal arch was expanded at a rate of 2 mm/month, for 2–8 months. The average treatment durations were 157.9 and 117.1 days, respectively, for the control and experimental groups. Dentoskeletal alterations were assessed on dental records, posteroanterior frontal cephalographs, and occlusal radiographs taken before and after treatment. Changes induced by treatments in each group and differences between changes in both groups were analyzed statistically (*α* = 0.05).

**Results:**

The treatment duration was significantly shorter in the experimental group (*P* < 0.05). The extent of dental displacement on the crossbite side was significant no matter what treatment was applied (*P* < 0.001); no between-group difference was detected (*P* > 0.05). Both treatments tilted the teeth in crossbite (*P* < 0.001) without any between-group difference (*P* > 0.05). The noncrossbite molar was displaced in the control group, whereas this did not occur in the experimental group (between-group *P* < 0.001).

**Conclusions:**

The Goshgarian TPA can be used with torque activation in order to deliver a more effective and faster correction of nonfunctional single-tooth unilateral crossbites with more favorable clinical results.

## 1. Introduction

A transpalatal arch (TPA) is a common orthodontic space management appliance used during the mixed or permanent dentitions [1–3]. It is a rigid wire passing a few millimeters over the palate and connecting to maxillary contralateral molars [2–4]. It may be used for various treatment goals such as stabilizing, anchorage, or for the movement of blocks of teeth in the horizontal, vertical, or sagittal planes; molar crossbites may be corrected, symmetric or asymmetric distalization of the maxillary molars may be facilitated, or buccal or lingual torque of their roots may be applied [1, 2, 5–10]. While the traditional form was a transpalatal bar extending over the palate to connect the first molars [11], the Goshgarian TPA is an alternative design incorporating an extra U-loop (in a forward or backward direction) in the middle of the arch [2, 4, 5, 12, 13].

Forces exerted by TPAs have been assessed in vitro [2, 3, 14, 15]. When activated symmetrically, the moments will be equal and in opposite directions, although the force might not be ideally symmetrical [2, 14]. Alternatively asymmetrical activation would lead to moments of different magnitude, causing different extents of tooth movement [2, 14].

Because they can move the maxillary molars, TPAs may be used for the treatment of dental crossbites [9, 10]. Unilateral crossbites are the most common forms of crossbite, and if not treated in an early stage, they may cause skeletal deformations [16–21]. The ideal form of correcting a unilateral lingual crossbite is to manipulate the affected molar buccally while maintaining the opposite molar (on the normal side) stationary [15, 22]. This may be achieved by buccal tipping of the affected molar against buccal torque applied to the anchorage tooth [15, 22]. While the effects of TPAs have been extensively studied, most have been in vitro or via finite element analysis [2, 3, 15]. The clinical literature on the treatment of posterior crossbites using a TPA is scarce and usually of low quality [9, 10]; and to the best of our knowledge, clinical studies on the correction of a unilateral crossbite using torque activation with a TPA is limited to one research [21]. Therefore, and considering the high prevalence of unilateral crossbites, this randomized clinical trial was conducted to compare the efficacy of two treatment methods “symmetric expansion of the arch versus arch expansion together with buccal torque activation of the molar roots” in treating patients having single-tooth nonfunctional dental posterior unilateral crossbites in the first permanent molar area. The null hypotheses were the lack of significant therapeutic effects of either method as well as the lack of any difference between the outcomes of both methods.

## 2. Subjects and Methods

This single-blind parallel randomized clinical trial examined 30 individuals with nonfunctional single-tooth unilateral crossbites in the first permanent molar area who were selected from local schools of three city districts as well as patients of the Orthodontic Department of Isfahan University. Subjects were screened according to the following eligibility criteria and identified as appropriate and invited for participation. The inclusion criteria were being between 6 and 16 years old and possessing a single-tooth unilateral nonfunctional crossbite in the first permanent molar area caused by local factors such as tooth malposition, without any mandibular deviation and shift. No patients had cleft lips/palates or any other craniofacial abnormalities. The patients could leave the study at any stage, and the treatment would still be provided to them completely. Written consent was taken from each subject and/or their parents after oral and written explanation. The study protocol was approved by the research committee of the university (t-4193/92072). The enrollment procedure took about 8 months to complete.

Initial treatment began with the correction of crossbite (without correcting other disorders) using TPAs only. Patients were randomly divided into two appliance groups: group 1 (control) included 15 patients (7 boys and 8 girls, Table 1) with an average age of 11.4 ± 2.1 years (range: 7.2 to 15.5), for whom a Goshgarian TPA was activated for simple symmetrical palatal expansion; and group 2 [experimental] included 15 patients (4 boys and 11 girls, Table 1) with an average age of 10.6 ± 1.8 years (range: 8.3 to 15.5) in whom the crossbite was corrected with a Goshgarian TPA activated to expand the palate in combination with buccal root torque on the normal side. The buccal root torque was delivered by twisting double-ended wire of TPA in the lingual direction and insertion in the lingual sheath, which was welded by the first author, before cementing the bands. The part of the double-ended wire on the crossbite side was cut off, so that the arm could rotate in the sheath [21]. Afterward, this torque was optimized by measuring the vertical distance between the occlusal surface of the first molar in the other side of the dental arch and TPA and confirming this distance to be around 10 mm. In the experimental group, the treatment was ended with overcorrection of the first molars, i.e., when the palatal cusp of the upper molars was facing the buccal cusp of the lower first molars. During the retention period (and after a slight relapse), a good cusp-to-groove occlusion was obtained. No bands were debonded during the study period. All the TPA appliances in both the groups were fabricated by the first author on the dental models and then activated before being inserted into the lingual sheaths. Fitting and adjusting of all appliances were done by the first author (senior orthodontics resident at the time of this research) and supervised by her professor.

The randomization was performed based on the order of registration, with patients alternately assigned to the control group or to the experimental group, respectively; the first patient was randomly assigned to the control group; the next and every other patient (subjects with even numbers) were assigned to the test group, while included participants with odd numbers were allocated to the control group. The orthodontist performing the treatments and measurements (who also randomized the groups) was aware of allocations. The patients were not aware (or informed of) their groups. They did not know the method by which their palatal arch was activated or the technical details of TPAs; therefore, without being informed by the orthodontist, they might not know the difference between treatments themselves; hence, the single-blind nature of this study.

**Figure 1:**
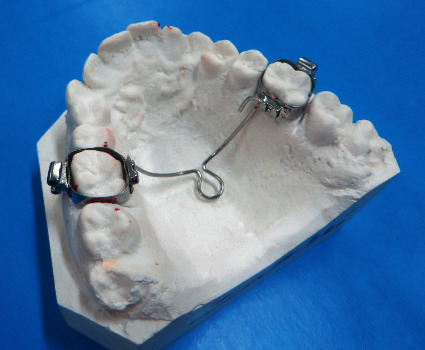
An inactive transpalatal arch.

**Figure 2:**
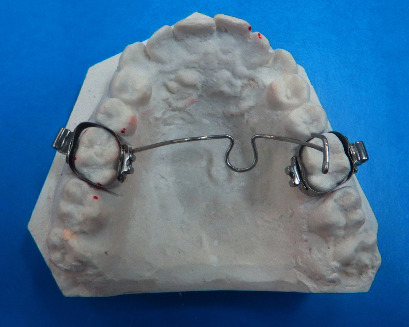
An activated transpalatal arch from the experimental group.

The TPA was universal and made of 0.036″ archwire, incorporating a U-loop in the center. The length of the palatal arch was determined by measuring the distance between contralateral maxillary first molars on dental casts, using a flexible ruler: The ruler was bent and held about 1 or 2 mm over the palate (Figure 1) [21]. This measurement was checked and verified in the mouth. The prepared wire was placed in the sheaths soldered to molar bands, and its passivity and relationship with the soft tissue was checked. The palatal arch was expanded for 2 mm/month, which was verified using an orthometer (Dentaurum, Ispringen, Germany). All TPAs were assessed monthly. At each examination session, each TPA was deactivated and then reactivated at the central loop (Figure 2). After treatment, inactive TPAs were maintained as retainers for three months.

**Figure 3:**
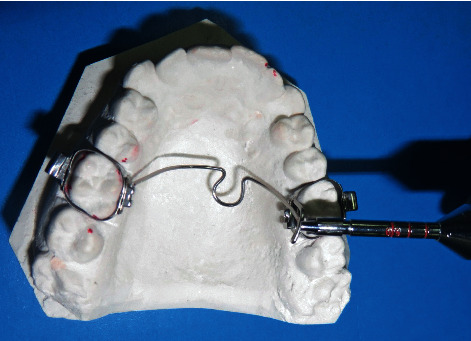
An example of force magnitude estimation.

At each session, an impression was taken and a new dental cast was poured. The force magnitude was measured at each treatment session, using a gauge (006-013-00; Dentaurum) on the dental cast and also in the mouth (as illustrated in Figure 3).

## 3. Records

Dental records, posteroanterior frontal cephalographs, and occlusal radiographs were taken before and after crossbite treatment. All radiographs were taken using the same unit under standard conditions (Planmeca, Helsinki, Finland). All films were coded by the clinician who performed the treatments but not the analysis.

Cephalographs were taken with Frankfurt plane positioned horizontally. All the measurements were measured twice (in the same session) by a researcher not blinded to treatments. The average of both measurements was calculated as the final values.

A bow divider was used to measure the distance between the central fossae of the right and left first maxillary molars as well as the distance between the midpalatal line and each of the left and right molars. These were performed before and after the treatment and were used to assess the displacement of the molars after the treatment. The extent of crossbite was only for the first permanent molars. The width of the anterior section was measured using occlusal reference points on the permanent canines (in 8 patients), the primary canines (in 7 patients), the primary first molars (in 2 patients), the permanent first premolars (in 8 patients), or the lateral incisors (in 5 patients). The use of different reference points was justified by the differences in the age of patients and their occlusal development.

**Figure 4:**
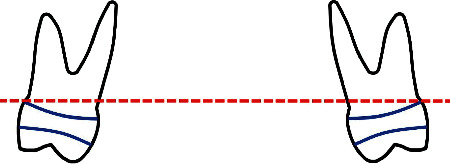
A schematic image of the frontal occlusal plane (the red dotted line).

Frontal posterior-anterior cephalographs were used to determine changes in the axes of the first molars after the treatment. Two planes were drawn over cephalographs to represent (1) the Frankfurt horizontal plane, which passed through the junction of the anatomic margin of the skull and external wall of the orbital fossa, and (2) the occlusal plane passing through the buccogingival edge of the molar bands (Figure 4). The angle between these two planes was measured before and after the treatment to show changes that happened to the occlusal plane after the treatment. The transverse axial inclination of the first molars was measured relative to the Frankfurt plane. The PA cephalographs were used for assessing buccal movements and torques of the first molars by measuring angular changes between the horizontal reference plane and the plane of metal rods attached to them.

In order to measure the inclination of the molars, a small metal rod was placed in the buccal tube in a way that the metal rod stood vertically and parallel to the buccal surface of the tooth. The angle between the image of this rod and the Frankfurt plane was measured using a cephalometric protractor with 0.5° accuracy (designed by Dr. A. T. Baum, 3M Unitek; Monrovia, CA, USA).

In accordance with the study of Ingervall et al. [21], occlusal radiographs were used to evaluate the effect of treatment on opening of the midpalatal suture as well as increases in palatal width.

## 4. Statistical Analysis

The sample size of this pilot study was determined as two groups of 15 patients, based on the literature [21]. The first and second primary outcomes were (1) changes happened by each treatment modality and (2) differences between the two methods, respectively. The Kolmogorov–Smirnov test was used for normality assessments. The groups' baseline conditions were compared using a Mann–Whitney *U* test and a Fisher exact test. The effects of treatment methods were analyzed using independent-samples and paired *t*-tests, a Wilcoxon test, and a Mann–Whitney *U* test of the SPSS program (SPSS Inc, Chicago, IL, USA). The level of significance was predetermined as 0.05.

## 5. Results

More than 50 patients were originally screened until reaching the desired sample size. After the inclusion, none of the subjects dropped out of the study. All patients were contacted frequently to ensure that they would attend all the sessions regularly. The baseline ages of both groups were not significantly different according to the Mann–Whitney *U* test (*P*=0.125). The Fisher test showed that there was not a significant difference between the groups in terms of the class (*P*=1.0), gender (*P*=0.450), and the crossbite side (*P*=0.715, Table 1). The trial ended when the desired sample size was reached. No major harms were identified during the study.

The mean treatment duration was 157.87 ± 56.66 days in the control group (range: 80 to 264 days), while it was 117.13 ± 44.35 days in the experimental group (range: 70 to 210 days). The treatment duration was significantly shorter in the experimental group compared with the control group (*P* < 0.05, *t*-test). The length of appliance loops was 45.33 ± 3.90 mm (range: 40 to 52) in the control group and 44.33 ± 5.42 mm (range: 36 to 55) in the experimental group, with no significant difference noted between the groups (*P* > 0.05). Force magnitudes were 248.00 ± 50.3 gr (range: 150 to 300) in the control group and 264.67 ± 52.22 gr (range: 200 to 400) in the experimental group, with no significant difference between the groups (*P* > 0.05). The crossbites were successfully treated in all patients.

### 5.1. Cast Analysis

The anterior width increased over time in each group after orthodontic treatment, without any significant difference apparent between the groups (Table 2).

The intermolar width showed significant changes caused by each treatment; the amounts of change were significantly different between the groups (*P* < 0.05).

The extents of dental displacement in the normal (noncrossbite) side differed significantly between the two groups (*P* < 0.01); in the experimental group, the tooth under buccal root torque showed no or minimal displacement (Table 2).

The amounts of dental displacement on the crossbite side were significant after either treatment (*P* < 0.001); however, the magnitudes of change did not differ significantly between the two groups (*P* > 0.05).

### 5.2. Radiographic Examination

Both treatment methods caused a significant change in the longitudinal axis of the teeth on the crossbite side (*P* < 0.001). However, there was no significant difference between the magnitudes of changes caused by either treatment (Table 2).

The extents of dental displacement in the normal (noncrossbite) side differed significantly between the two groups (*P* < 0.001). In the experimental group, the tooth under buccal root torque showed no or minimal displacement (Table 2).

The occlusal plane showed minimal or no change after the treatment (Table 2). The midpalatal suture failed to open.

## 6. Discussion

TPAs are clinically favorable because they distribute the force over most of the teeth and reduce the force exerted on a single tooth. This prevents tooth luxation and patient pain or discomfort compared with the process of rapid palatal expansion [22]. There are differences in the treatment of unilateral functional and nonfunctional crossbites. For instance, functional posterior crossbites can be treated mainly by occlusion adjustments, while TPAs are needed for treatment of nonfunctional crossbites [9, 10, 23].

The findings of the present study indicated that treatment using Goshgarian TPAs, which exerted buccal root torque on the normal side, led to faster and more effective corrections compared with symmetrical palatal expansion. The force exerted in the torque activation group was slightly and insignificantly greater than that in the symmetrical expansion group. Both treatments increased the anterior arch width similarly. However, the intermolar width increased to a significantly greater extent in the symmetric expansion group, which could be attributable to buccal displacement of both sides [21]. A comparison of the distance between the teeth and the midpalatal raphe indicated a similar extent of tooth displacement on the affected side, in both groups. However, on the normal side, the extent of molar displacement in the torque activation group was minimal and much smaller than that seen in the symmetric expansion group. On the affected side, both treatments tilted the molars significantly, and to a similar extent (about 13° to 14°). Only the symmetric expansion method tilted the molars on the normal side, while the torque activation method barely tilted the molars on the normal side. These findings were mostly in line with the only published clinical report and suggest the torque activation method as a viable alternative to conventional symmetrical TPA treatment [21]. Anterior displacement of the maxillary molars results in their mesiolingual rotation around their palatal root, narrowing the space between the cortical and buccal and lingual plates anterior to the roots of the first molar; this prevents the direct advancement of the molars and constrains its displacement to a rotation [4, 11]. Since the palatal root engages with the lingual plate, the buccal roots rotate mesiolingually. Moreover, coupling the left and right molars prevents any rotations and reduces the anterior movement [4].

The differences between this study and that of Ingervall et al. [21] were related to the intermolar width, which was not found to be significantly different between the two methods [21]. Moreover, Ingervall et al. [21] reported a significant difference between molar displacement on the crossbite side, while in the present study, both groups tipped their molar crowns to a similar extent (about 3.9 mm compared to 4.2 mm). Finally, Ingervall et al. [21] reported a slight opening in the midpalatal suture (which was more noticeable in the torque activation group), while no separation was observed in the present research. The differences may be due to methodological variations such as the patients' ages or statures, or the magnitude or duration of force delivery.

Although torque activation is a viable stabilizing method, there may be side effects. The force moment exerting on the anchorage molar should be balanced by the vector of vertical forces (ranging from 50 to 120 g), which might extrude the molar in crossbite and intrude the contralateral molar [15, 21, 24]. The clinical outcome of the vertical forces was investigated in the present study through an assessment of the transverse occlusal plane. The inclination of the occlusal plane barely changed as a result of either treatment (about 0.5° in the experimental group and 0.6° in the control group) without any significant between-group differences. This inclination was possibly because of the tipping of the molar crowns (as the reference points of the occlusal plane), which would alter their vertical and horizontal positions in the transverse plane [21]. This magnitude of change was significant only in the control group and not in the torque activation group. The findings of the present study were similar to those of Ingervall et al. [21] who reported slight and similar changes of the transverse occlusal plane inclination in both groups without any serious extrusion of the molars on the crossbite side in their torque activation group [21]. The only difference between the studies was that Ingervall et al. [21] found that the altered occlusal inclinations after both treatments were statistically significant, while in the present study, the inclination observed in the torque activation group did not reach a level of significance. TPAs should remain passive while being cemented, in order to prevent transverse or vertical displacements of the permanent molars [4, 25]. There are a number of ways to determine the occlusal plane in frontal/coronal aspect. Some authors define it as the line intersecting the occlusal surfaces of the first molars or canine cusp tips [26, 27]. However, Ingervall et al. [21] defined the occlusal plane as a line intersecting the gingival edges of the bands on the buccal sides of the first molars. Since their study [21] was the most relevant one to ours, we as well used their definition.

This study was limited by some factors. For randomization, more advanced methods such as random generators or random number tables should be used. Still, the current method was completely random because the first assignment was random and the subsequent inclusion of patients into the study was completely out of the control of the authors or patients and following a random pattern. A larger sample based on power calculations could favor the reliability. However, this was a preliminary study itself, and its results can be used for future power and sample size calculations. Furthermore, the current sample size sufficed for detection of numerous significant results. Another limitation was the use of radiography in this research, which can raise ethical concern due to hazardous X-ray nature. However, most of the radiographs taken were routine clinical procedures, and the occlusal radiographs taken in addition to clinical procedures were in accordance with the previous study [21]. Manual tracing on papers might not be as fast and accurate as digital tracing (along with digital radiography) [28, 29]; nevertheless, accuracies of both methods have been found to be similar [30]. The inclusion criteria were determined as between 6 and 16 years of age. Therefore, all prepubertal, pubertal, and postpubertal children on different skeletal maturational stages were included, whose response to the same treatment protocol might inevitably be different by dental, alveolar, and skeletal means, and even in some of them, the palatal suture might have opened. This is such a wide age range and can disrupt the uniformity of the sample. Nevertheless, despite all the age diversity, the sample size and the contrasts sufficed to detect statistically significant differences. Also having a broader age range can improve the generalizability. Still, future studies should assess this method for each of the age ranges and growth stages. Furthermore, intra- or interobserver reliability analyses were not done in this study. Additionally, it was better to use anatomic landmarks to determine the occlusal plane. Still, the orthodontic bands had been selected carefully under the supervision of an experienced orthodontist, and all had been placed very carefully in proper positions. Moreover, again all the palatal sheaths had been welded to the bands at proper heights. The palatal arch was placed within the sheaths and removed before activating and afterward was placed into the sheaths again. Therefore, the bands underwent no noticeable dislocations or cement loosening. The obtained statistical significance as well implies the uniformity of the measurements at both sessions (and hence implies a lack of displacements).

## 7. Conclusion

The findings of this pilot randomized clinical trial suggest that a Goshgarian TPA might be used with torque activation in order to deliver a faster correction of single-tooth unilateral nonfunctional posterior dental crossbite in the first permanent molar area with appropriate clinical results comparable with those of conventional symmetrical expansion and without adverse effects beyond those of the conventional method (i.e., no extra inclination of transverse occlusal plane, no or minimal displacement of the anchorage molar).

## Figures and Tables

**Table 1:** Sample characteristics.

Group	Crossbite side	Occlusion	Sex	Age (Years ^Months^)
*Group 1-Control (symmetrical expansion)*	Right	CL I	Male	10^6^
	Left	CL I	Female	11
	Right	CL II, SUB, R	Male	9^3^
	Left	CL I	Female	11^2^
	Right	CL I	Male	10^7^
	Left	CL I	Male	13^2^
	Left	CL I	Male	12^4^
	Left	CL II	Female	15^2^
	Right	CL I	Female	11
	Left	CL I	Female	7^2^
	Left	CL I	Male	15^6^
	Right	CL I	Female	9^5^
	Right	CL I	Female	11^3^
	Right	CL I	Female	11^6^
	Right	CL I	Male	11^7^

*Group 2-Experimental (torque activation)*	Right	CL I	Male	8^6^
	Left	CL I	Female	10^2^
	Right	CL I	Female	8^3^
	Left	CL I	Female	10^4^
	Right	CL I	Female	9^8^
	Left	CL I	Male	11^5^
	Right	CL I	Female	10^3^
	Left	CL I	Male	10^6^
	Left	CL I	Female	9^4^
	Left	CL I	Female	15^6^
	Right	CL I	Female	11^5^
	Right	CL I	Male	11
	Left	CL I	Female	13^2^
	Left	CL II	Female	10^1^
	Left	CL I	Female	9^3^

**Table 2:** Post-treatment changes in each group (i.e., changes occurred during treatment and measured after it), and the comparison of change extents in both groups.

Parameters	Control (symmetric expansion)						Experimental (torque activation)						BG Diff
	Mean	SD	Min	Max	95% CI		Mean	SD	Min	Max	95% CI		
Anterior arch width (mm)	1.20^*∗∗∗*^	1.01	0	3	0.64	1.76	1.07^*∗∗∗*^	0.70	0	3	0.68	1.46	*P* > 0.05
Intermolar width (mm)	6.13^*∗∗∗*^	1.96	3	9	5.04	7.22	4.77^*∗∗∗*^	1.03	3	6	4.20	5.34	*P* < 0.05

*Molar-Raphe distance (mm)*													
Crossbite side	3.90^*∗∗∗*^	1.26	2.5	7	3.20	4.60	4.17^*∗∗∗*^	1.45	1	6.5	3.37	4.97	*P* > 0.05
Noncrossbite side	2.23^*∗∗∗*^	1.52	0.5	6	1.39	3.07	0.63^*∗*^	1.23	−2	2.5	−0.05	1.31	*P* < 0.01

*Molar inclination (°)*													
Crossbite side	13.13^*∗∗∗*^	7.12	1	28	9.19	17.07	14.17^*∗∗∗*^	7.99	3	26.5	9.75	18.59	*P* > 0.05
Normal side	12.93^*∗∗∗*^	5.91	3.5	24	9.66	16.20	0.67	5.35	−8	9.5	−2.29	3.63	*P* < 0.001
Occlusal plane inclination (°)	0.63^*∗*^	1.37	−1	3	−0.13	1.39	0.47	1.23	−1	3	−0.21	1.15	*P* > 0.05

SD, standard deviation; Min, minimum; Max, maximum; CI, confidence interval; BG Diff, between-group difference (difference between the extents of changes caused by treatments in the two groups, calculated using the independent-samples *t*-test or Mann–Whitney). The lack of asterisks over mean values indicates that the treatment did not cause significant changes to the parameter (comparing pre- and post-treatment measurements in each group). The presence of asterisk(s) indicates that the extent of within-group change (happened during treatment and measured after it) was significant compared to the baseline: ^*∗*^*P* < 0.05 and ^*∗∗∗*^*P* < 0.001 (calculated using the paired *t*-test or its nonparametric alternative, the Wilcoxon test).

## Data Availability

The raw data are available from the authors.
